# The angiotensin‐converting enzyme insertion/deletion polymorphism rs4340 associates with habitual physical activity among European American adults

**DOI:** 10.1002/mgg3.308

**Published:** 2017-07-02

**Authors:** Michael Bruneau, Theodore J. Angelopoulos, Paul Gordon, Niall Moyna, Paul Visich, Robert Zoeller, Rick Seip, Stephen Bilbie, Paul Thompson, Joseph Devaney, Heather Gordish‐Dressman, Eric Hoffman, Linda S. Pescatello

**Affiliations:** ^1^ Drexel University Philadelphia Pennsylvania; ^2^ Emory & Henry College Emory Virginia; ^3^ Baylor University Waco Texas; ^4^ Dublin City University Dublin Ireland; ^5^ University of New England Biddeford Maine; ^6^ Florida Atlantic University Boca Raton Florida; ^7^ Hartford Healthcare Hartford Connecticut; ^8^ Children's National Medical Center Washington District of Columbia; ^9^ Cooperative International Neuromuscular Research Group Washington District of Columbia; ^10^ University of Connecticut Storrs Connecticut; ^11^ University of Connecticut Institute for Systems Genomics Storrs Connecticut

**Keywords:** Body mass index, exercise, genes, renin‐angiotensin aldosterone system

## Abstract

**Background:**

The angiotensin‐converting enzyme (ACE) insertion/deletion (I/D) polymorphism (rs4340) (*ACE DIP)* accounts for half of the variability in plasma ACE concentrations. *ACE* has been widely studied for its influence on sports performance; however, research on its influence in physical activity is limited and inconsistent. We examined the influence of the *ACE DIP* on physical activity among 461 European Americans.

**Methods:**

Subjects completed the Paffenbarger Physical Activity Questionnaire for weekly walking distance. Multivariate analysis of covariance (MANCOVA) tested log‐transformed differences in weekly walking distance among *ACE DIP* genotypes (II, ID, DD) with gender as a fixed factor, and age and body mass index (BMI) as covariates. Because we found a significant *ACE DIP*xBMI interaction (*P* = 0.03), we categorized the sample by normal weight (NW: BMI<25.0 kg/m^2^) and overweight (OW: BMI ≥25.0 kg/m^2^) and repeated the MANCOVA with multiple comparison adjustments.

**Results:**

NW adults with *ACE* II walked 15.8 ± 11.1 km/week, ID 13.2 ± 10.6 km/week, and DD 17.9 ± 13.0 km/week, with ID walking less than II (*P* = 0.03) and DD (*P* = 0.01). OW adults with *ACE* II walked 16.7 ± 12.6 km/week, ID 13.8 ± 11.6 km/week, and DD 9.7 ± 9.0 km/week, with DD walking less than II (*P* = 0.02). Weekly walking distance was 8.2 ± 2.4 km/week less among OW adults with *ACE* DD than NW (*P* = 0.02).

**Conclusion:**

BMI interacted with *ACE* DD such that OW walked ~8.2 km/week less than NW, potentially equating to a body weight differential of ~3.5 kg annually.

## Introduction

The angiotensin‐converting enzyme (ACE) is a biological catalyst for the conversion of angiotensin I to angiotensin II, a potent vasoconstrictor in the renin‐angiotensin aldosterone system (RAAS). Angiotensin II stimulates the release of aldosterone and antidiuretic hormone, actions which increase renal sodium and water reabsorption, stimulate thirst, and modulate sympathetic vascular tone. The ACE insertion (I) deletion (D) polymorphism (*ACE DIP*; OMIM +106180; GenBank KJ140509.1) (rs4340) is a 287‐base pair sequence within intron 16 of chromosome 17q23 that accounts for half of the variability in plasma ACE concentrations (Cambien et al. 1988; Wong et al. 2012). Those homozygous for the D allele of the *ACE DIP* exhibit higher serum ACE and angiotensin II levels and less bradykinin levels, a vasodilator, than those homozygous for the I allele of the *ACE DIP* (Wong et al. 2012).

Due to its influence on cardiovascular and renal function, the *ACE DIP* has been examined extensively as a polymorphism that influences sports performance as well as the response of health‐related phenotypes to acute (i.e., short‐term) and chronic (i.e., long‐term or training) exercise (Hagberg et al. 1998; Woods et al. 2000a,b; Zhang et al. 2003; Jones et al. 2002; Jones and Woods 2003; Williams et al. 2000; Rankinen et al. 2000; Pescatello et al. 2006; Kostis et al. 2002). Those homozygous for the I allele of the *ACE DIP* exhibit higher maximal and submaximal oxygen consumption (Hagberg et al. 1998; Woods et al. 2000a,b), a greater propensity of type 1 muscle fibers (Zhang et al. 2003), and enhanced substrate utilization for long‐term and sustained aerobic activities (Jones et al. 2002; Jones and Woods 2003; Williams et al. 2000). In contrast, those homozygous for the D allele of the *ACE DIP* exhibit a higher propensity of type 2 muscle fibers and perform better at strength and anaerobic sport activities (Zhang et al. 2003).

The literature regarding the association of the *ACE DIP* with habitual physical activity is much more limited (Wong et al. 2012; Hagberg et al. 1998; De Moor et al. 2009; Fuentes et al. 2002; Winnicki et al. 2004). Those homozygous for the I allele of the *ACE DIP* have been reported to engage in higher levels of habitual physical activity than those homozygous for the D allele of the *ACE DIP* (Wong et al. 2012; Winnicki et al. 2004), but this finding has been inconsistent across studies (Hagberg et al. 1998). Reasons for this discrepant literature are not clear, but may be due to a limited number of investigations that had small heterogeneous samples that may have been underpowered, and/or lacked statistical control for potential confounders that would influence possible associations with the *ACE DIP* and habitual physical activity such as age and body mass index (BMI) (Wong et al. 2012; Hagberg et al. 1998; De Moor et al. 2009; Fuentes et al. 2002; Winnicki et al. 2004). The purpose of our study was to examine associations of the *ACE DIP* with habitual physical activity among a large sample of 461 European American adults from the *Functional Single Nucleotide Polymorphisms Associated with the Human Muscle Size and Strength* (FaMuSS, NIH R01 NS40606‐02) study. We hypothesized that those homozygous for the I allele of the *ACE DIP* would report higher levels of habitual physical activity compared to those homozygous for the D allele.

## Methods

### Ethical compliance

The FaMuSS study was a multi‐center trial conducted by the Exercise and Genetics Collaborative Research Group consisting of researchers from the University of Central Florida, University of Massachusetts, West Virginia University, Dublin City University, University of Connecticut, Central Michigan University, Florida Atlantic University, Yale University, Hartford Hospital, and the Children's National Medical Center. The institutional review boards from all 10 institutions approved the study protocol. The primary aim of FaMuSS was to identify genetic factors that dictated the response of health‐related fitness phenotypes to resistance exercise training (RT). Prior to RT, investigators obtained blood samples for determination of cardiometabolic profiles and DNA extraction, and assessed habitual physical activity with the Paffenbarger Physical Activity Questionnaire. The experimental design of FaMuSS has been described elsewhere, so only the methods related to this substudy are described (Pescatello et al. 2013).

### Subjects

All subjects provided written informed consent. Subjects were excluded if they: (1) had performed RT within the past year; (2) had a chronic health condition that would preclude their ability to perform RT; (3) had metal implants in the arms, eyes, head, brain, neck, and/or heart that would be contraindicated to magnetic resonance imaging (MRI); (4) were prescribed and/or taking medications (i.e., corticosteroids, anabolic steroids, antihypertensive or antilipidemic medications, diuretics, Depo‐Provera contraceptive injection, Clenbuterol, Rhinocort nasal inhaler, lithium nonsteroidal anti‐inflammatory medications) known to effect skeletal muscle function; (5) consumed an average of ≥2 alcoholic drinks per day; (6) consumed dietary supplements to enhance muscle strength and size or weight; or (7) gained or lost >2.2 kg of body weight within 3 months prior to enrolling in the study. In all, 461 European American adults (≥18 years) were genotyped for the *ACE DIP* (rs4340) and comprised the sample for this substudy.

### Physical activity

Upon enrollment into FaMuSS, subjects were measured for body weight (kg) and height (cm) to determine BMI (kg/m^2^). Subjects then completed the Paffenbarger Physical Activity Questionnaire to assess weekly habitual physical activity over the past year, including informal activities of daily living (e.g., city blocks walked and stairs climbed), leisure time activities, and formal exercise (Paffenbarger et al. 1978). Physical activity scores in kcal/week were calculated based on the sum of calories expended across durations and intensities of the different activities reported. The Paffenbarger Physical Activity Questionnaire has excellent reliability and predictive validity and is sensitive to changes to interventions (Pereira et al. 1997; Harris et al. 1994; Jeffery et al. 1998). The physical activity phenotype examined in this substudy was derived from the following question: “How many city blocks or their equivalent did you walk on an average day during the past year?”. The self‐reported weekly walking distance in kilometers (km) per week was totaled and used for comparison among the *ACE DIP* genotypes.

### Genotyping

Fasting blood samples (21 mL) were taken from each subject in standard EDTA vacutainer tubes (Becton Dickson and Company, Franklin Lakes, NJ, USA). Serum blood samples were centrifuged at 4°C and were spun at 1100 × *g* per min for 10 min. Hemolysized samples were redrawn as a quality control measure. The samples were then refrigerated and stored at −80°C until being transported to the Children's National Medical Research Center in Washington, DC. DNA was isolated from peripheral blood lymphocytes with a Gentra Puregene DNA extraction kit (Qiagen, Valencia, CA, USA) and *ACE DIP* (rs4340) genotypes (II, ID, DD) were determined with TaqMan allele discrimination assays employing a 5’ nuclease activity of Taq polymerase to detect a fluorescent reporter signal generated during polymerase chain reactions. Endpoint fluorescent readings for *ACE DIP* genotypes were then performed with a standard ABI 7900HT Sequence Detection System (SDS V 2.3 software; Applied Biosystems, Foster City, CA, USA). All genotyping was performed in duplicate as a quality control measure and any disagreements in genotype readings were repeated.

### Statistical analysis

Descriptive statistics were calculated as means ($\overset{¯}{X}$) ± standard deviations (SD) for all variables, unless otherwise noted. Chi‐square analysis was used to confirm that the *ACE DIP* (rs4340) was in Hardy–Weinberg equilibrium (HWE) with genotype frequencies of 25.8% (II), 46.2% (ID), and 28.0% (DD). Multivariate analysis of covariance (MANCOVA) was used to test log‐transformed differences in weekly walking distance among *ACE DIP* genotypes (II, ID, DD) with gender as a fixed factor and age and BMI as covariates. Because a significant *ACE DIP*xBMI interaction was found (*P* = 0.03), we categorized the sample into normal weight (NW: BMI ≤24.9 kg/m^2^) and overweight (OW: BMI >24.9 kg/m^2^) groups. We then repeated the MANCOVA with gender and BMI groups as fixed factors and age as a covariate. Post hoc univariate ancovas and subsequent pairwise comparisons were assessed with simple effects tests and multiple comparison adjustments with corrected alpha levels set at *P* < 0.02. All analyses were performed using the Statistical Package for the Social Sciences (SPSS, Armonk, NY, USA) 21.0.

## Results

### Subject characteristics

The sample included 461 European Americans who on average were young, normal weight (*n* = 307, BMI = 21.7± 1.8 kg/m^2^) or overweight (*n* = 154, BMI = 29.6± 4.4 kg/m^2^) men (*n* = 203, 44%) and women (*n* = 258, 56%) (Table 1). Subject physical characteristics did not differ by *ACE DIP* (rs4340) genotype (*P* > 0.05), but did differ by gender for height, weight, and BMI (*P* < 0.05). When the sample was divided by BMI as NW or OW groups, OW subjects were older than NW subjects (*P* < 0.05) (Table 2).

**Table 1:** Mean (±SD) physical characteristics of the total sample and by *ACE DIP* (rs4340) genotype

Genotypes				
Variable	Total sample (*n* = 461)	II (*n* = 119)	ID (*n* = 213)	DD (*n* = 129)
Age (year)	24.1 ± 15.7	23.8 ± 15.3	24.2 ± 15.9	24.2 ± 15.8
Weight (kg)	70.5 ± 16.5	70.5 ± 14.8	71.0 ± 17.0	69.7 ± 17.3
Height (cm)	169.7 ± 19.2	170.1 ± 18.4	169.7 ± 19.6	169.5 ± 19.2
BMI (kg/m^2^)	24.3 ± 14.8	24.3 ± 14.4	24.6 ± 15.0	24.1 ± 14.7

There were no significant baseline differences in the physical characteristics of subjects among *ACE DIP* genotypes (*P* > 0.05). *ACE,* angiotensin‐converting enzyme; I, insertion allele; D, deletion allele; BMI, body mass index.

**Table 2:** Mean (±SD) physical characteristics by BMI group

Variable	NW (*n* = 307)	OW (*n* = 154)
Age (year)	23.2 ± 6.4	25.8 ± 16.4^a^
Weight (kg)	62.3 ± 8.4	87.0 ± 16.4^a^
Height (cm)	169.1 ± 8.8	171.0 ± 19.8^a^
BMI (kg/m^2^)	21.7 ± 1.8	29.6 ± 14.4^a^

a *P* < 0.05, NW versus OW. BMI, body mass index; NW, normal weight; OW, overweight.

### ACE DIP genotypes and weekly walking distance by BMI group

**Figure 1:**
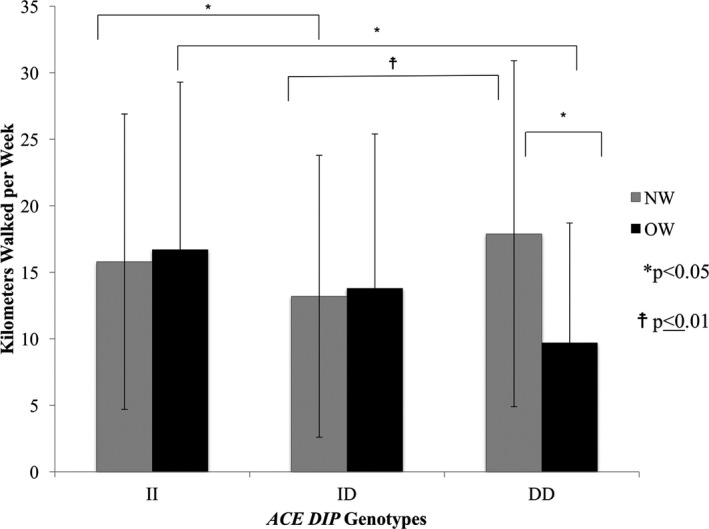
*ACE DIP* genotypes and weekly walking distance by BMI group.

For NW, subjects with *ACE* II walked 15.8 ± 11.1 km/week, ID 13.2 ± 10.6 km/week, and DD 17.9 ± 13.0 km/week, with ID walking 2.6 ± 1.4 km/week less than II (*P* = 0.03) and 4.7 ± 1.6 km/week less than DD (*P* = 0.01). For OW, subjects with *ACE* II walked 16.7 ± 12.6 km/week, ID 13.8 ± 11.6 km/week, and DD 9.7 ± 9.0 km/week, with DD walking 7.1 ± 3.7 km/week less than II (*P* = 0.02). When the NW and OW groups were directly compared, weekly walking distance was 8.2 ± 2.4 km/week less among OW than NW with *ACE* DD (*P* = 0.02) (Fig. 1).

### ACE DIP genotypes and weekly walking distance by body weight classification and gender

NW men with the II genotype reported walking 0.8 ± 2.6 km/week more than NW women with the II genotype (*P* = 0.02). No other genotype by physical activity phenotype associations were found among *ACE DIP* genotypes, gender, or body weight classification (*P* ≥ 0.05).

## Discussion

The purpose of this FAMuSS substudy was to determine whether the *ACE DIP* (rs4340) was associated with weekly walking distance in a large sample of healthy European American adults. The primary finding was that the *ACE* DD genotype interacted with BMI such that OW adults walked, on average, 8.2 km/week less than NW adults. We also found NW adults with ID walked 2.6 km/week less than II and 4.5 km/week less than DD; whereas, OW adults with DD walked 7.1 km/week less than II.

Our findings are consistent with those of Wong et al. who found that subjects with the *ACE* D allele had lower levels of physical activity than those with the I allele among a sample of 110 Chinese adults with normal blood pressure. More specifically, they found that those with the *ACE* DD genotype expended 1232 fewer kilocalories and had a 6.88 greater likelihood of engaging in either insufficient or low levels of physical activity compared to those with the II genotype (Wong et al. 2012). Similarly, Winnicki et al. (2004) investigated the association of the *ACE DIP* and physical activity status among 355 Caucasians with untreated hypertension and found 76% of the sample with the *ACE* DD and 62% with ID genotypes were sedentary compared to 48% with the II genotype.

The exact mechanisms to explain the differential effects of the *ACE DIP* on habitual physical activity levels are unclear. However, several biologically plausible explanations exist that support our findings. First, Wong and colleagues theorized that a possible explanation for lower physical activity levels among those with the *ACE* D allele could be attributed to higher serum ACE concentrations that result in a greater rate of conversion of angiotensin I to angiotensin II, a potent vasoconstrictor that inhibits the production of bradykinin, a potent vasodilator. Previous in vivo evidence by Stebbins and Longhurst has supported this proposed mechanism as small doses of bradykinin injected into the skeletal muscle of cats was found to initiate a reflexive change in cardiovascular hemodynamics, similar to that induced by physical activity and exercise (Stebbins and Longhurst 1985). As a result of these vascular alterations, Wong et al. speculated that participation in habitual physical activity may be perceived as more difficult and less desirable to perform in adults with the *ACE* D allele compared to those with the I allele due to a reliance on glycolytic energy pathways from a diminished oxygen availability in the exercising muscles. In support of their premise are the findings of Thompson et al. (2006) who examined the influence of the *ACE DIP* on adherence to an endurance exercise training intervention among 110 healthy men and women and found adherence rates that were 2% and 6% lower for the ID and DD genotypes compared to the II genotype.

Alternatively, those with the *ACE* DD genotype have been shown to possess fewer than 20% of their muscle fiber types as type 1 (Zhang et al. 2003). Type 1 fibers are better suited for endurance physical activity such as walking than type 2 fibers that are better suited for resistance physical activity. Although in vivo evidence of type 1 fibers and habitual physical activity has been limited by a lack of investigation, animal models bred for low aerobic performance have shown a smaller proportion of type 1 muscle fibers that were more sensitive to fatigue and had a slower metabolic recovery from electrically stimulated exercise (Torvinen et al. 2012). In accordance with these findings, Thompson et al. postulated that endurance exercise may be more preferable for those with the *ACE* I allele than those with the D allele due to differences in muscle fiber type composition, a postulation that is also consistent with our findings and those of Wong et al. of lower physical activity levels in those with the *ACE* DD than II genotype (Wong et al. 2012; Thompson et al. 2006).

Evidence for the biological plausibility of our major finding of OW walking 8.2 km/week less than NW is lacking. We propose that differences in muscle fiber type and/or serum ACE levels may account for possible interactions between the *ACE DIP* and the BMI groups that we observed. Previous research by Tanner et al. comparing the muscle fiber types of 53 women found lesser propensities of type 1 and greater propensities of type 2 muscle fibers in women with obesity (Tanner et al. 2001). Differences in serum ACE levels have also been found between NW and OW with the *ACE* DD genotype. ACE is a membrane‐based metallopeptidase located in vascular endothelial cells that functions as a biological catalyst in the RAAS. Cooper et al. examined the relationship between the RAAS and obesity in 500 Jamaican adults and found higher serum ACE levels among adults with obesity (Cooper et al. 1997). Furthermore, Rigat et al. (1990) explored the relationship between the *ACE DIP* and serum ACE in 80 non‐Hispanic White adults and found higher serum ACE levels among adults with the DD genotype compared to either the II or ID genotypes. Having a greater preponderance of type 2 muscle fiber types and/or higher serum ACE levels among adults with OW and the *ACE* DD genotype could account for our finding of walking 8.2 km/week less than adults with NW and *ACE* DD due to their higher levels of adiposity. However, since we did not measure ACE nor determine muscle fiber type, further research is needed to determine if our supposition is true.

The present substudy is not without limitation. Because the primary purpose of FaMuSS was to examine the influence of genetic variation on human muscle size and strength in response to RT, this substudy was not designed to examine habitual physical activity as primary outcome measure. We assessed physical activity with the Paffenbarger Physical Activity Questionnaire, a well validated and reliable tool used for the assessment of leisure time physical activity in similar populations to the population used in FaMuSS. However, the self‐reported recall of habitual physical activity from the previous year may have been limited by inaccuracies in subject reporting and/or social desirability bias (Paulhus 1991; Chastin et al. 2014). We also used BMI as a surrogate measure of body fat to segregate our subsample into NW and OW groups, which does not account for differences in muscle mass nor muscle fiber type between groups (Romero‐Corral et al. 2008). Finally, our substudy was not mechanistic in nature, so we can only speculate on the reported mechanisms that may explain our findings.

Despite these limitations, our substudy has several important strengths. To date, the literature regarding the association of the *ACE DIP* with habitual physical activity has been limited due to a small number of investigations that have included small and heterogeneous samples that were underpowered to detect statistical associations between the *ACE DIP* and physical activity. Our substudy attempted to address these limitations by including a large sample of 461 healthy European American adults that was sufficiently powered to detect our observed effect size of 0.11 with 83% power at an alpha level set to *P* < 0.05 (Faul et al. 2007). Our substudy also controlled for the potential confounding variables of age and BMI group, which could have influenced the associations found between the *ACE DIP* and weekly walking distance.

## Conclusion

In summary, we found the *ACE DIP* (rs4340) was associated with habitual physical activity in a large sample of European American adults from FaMuSS. OW adults with the DD genotype walked an average of 8.2 km/week less compared to NW adults with this genotype. This finding is novel and has clinical significance that could potentially equate to a body weight differential of ~3.5 kg per year. Future experimental studies are needed to confirm our findings and investigate possible mechanisms for the *ACE DIP*xBMI interaction we observed with more objective assessments of physical activity.

## Conflict of Interest

The authors declare no conflict of interest with this manuscript.
